# Acute postoperative pain trajectories and their impact on functional recovery following total knee arthroplasty

**DOI:** 10.3389/fpain.2025.1659917

**Published:** 2025-10-13

**Authors:** Caijin Wen, Qin Qin, Lu Wei, Xi Luo, Jing Zhang

**Affiliations:** ^1^ School of Nursing, North Sichuan Medical College, Nanchong, Sichuan, China; ^2^ Nursing Department, Panzhihua University Affiliated Hospital, Panzhihua, Sichuan, China; ^3^ Orthopedics Department, Panzhihua Central Hospital, Panzhihua, Sichuan, China

**Keywords:** total knee arthroplasty, acute postoperative pain, growth mixture model, joint functional outcomes, influencing factors

## Abstract

**Objective:**

To investigate the trajectories of acute postsurgical pain (APSP) following total knee arthroplasty (TKA), its influencing factors, and its impact on knee function recovery at 3 months postoperatively.

**Methods:**

A convenience sample of patients undergoing TKA at a tertiary hospital in Panzhihua City between June 2024 and February 2025 was recruited. Preoperatively (T0), baseline data including demographics, anxiety, depression, family care index, pain level, and pain catastrophizing were collected. Postoperative pain levels were assessed on days 1 (T1), 2 (T2), 3 (T3), and 5 (T4), while joint functional outcomes were evaluated at 3 months postoperatively (T5). Growth mixture modeling (GMM) was used to identify distinct APSP trajectory subgroups, logistic regression was used to analyze influencing factors, and multiple linear regression was used to examine the association between APSP trajectories and joint functional outcomes.

**Results:**

Among 227 enrolled patients, two APSP trajectory subgroups were identified: a moderate-high persistent pain group (45.16%) and a moderate-low rapid relief group (54.84%). Logistic regression revealed that age, preoperative pain level, pain catastrophizing, and family care index significantly influenced APSP trajectories. APSP trajectory membership positively predicted 3-month knee joint functional outcomes.

**Conclusion:**

TKA patients exhibit two distinct APSP trajectory patterns, which serve as significant predictors of joint functional outcomes. Clinicians should identify the persistent pain subgroup and implement enhanced multimodal analgesia to prevent chronic postsurgical pain and optimize rehabilitation outcomes.

## 1 Introduction

Total Knee Arthroplasty (TKA) is a pivotal intervention for end-stage knee pathologies, effectively alleviating pain, restoring function, and correcting deformities (1). Pain, as one of the most critical perioperative concerns in orthopedic patients, ranks second in patient-reported outcomes (PROs) (2). Studies indicate that among patients dissatisfied post-TKA, 39% attribute their dissatisfaction to pain-related factors (3). Postoperative pain can be categorized into acute, subacute, and chronic based on duration. Acute postsurgical pain (APSP), a hallmark of surgical stress response, exhibits a characteristic temporal pattern, peaking within 24–72 h postoperatively and typically persisting for 4–6 days (4). Notably, APSP occurs in nearly 100% of TKA patients. Under the Enhanced Recovery After Surgery (ERAS) protocol, early mobilization is essential, yet movement-associated pain remains a key barrier to rehabilitation. Longitudinal studies by Puolakka et al. (5) further demonstrate that APSP intensity within the first postoperative week significantly correlates with the development of chronic postsurgical pain (CPSP). Such persistent pain not only impedes functional recovery but may also trigger psychological comorbidities (e.g., anxiety, depression), ultimately impairing health-related quality of life (HRQoL) across multiple domains. While international research has systematically mapped subacute and chronic pain trajectories post-TKA, investigations into acute-phase pain evolution remain preliminary (6–9). Although studies (10–13) confirm the temporal dynamics and individual heterogeneity of APSP after TKA, many rely on mixed-surgical cohorts, obscuring TKA-specific pain mechanisms. Moreover, the relationship between APSP trajectories and long-term PROs remains unexplored. So, this prospective cohort study employs a growth mixture model (GMM) to (1) delineate APSP trajectories in TKA patients, identifying distinct pain-pattern subgroups and their predictors; and (2) evaluate the impact of these trajectories on 3-month postoperative functional recovery. The findings aim to guide personalized pain management strategies and improve clinical outcomes.

## 2 Subjects and methods

### 2.1 Study participants

Using a convenience sampling approach, we enrolled patients undergoing TKA at a tertiary Grade-A general hospital in China between June 2024 and February 2025. Inclusion criteria: Age ≥18 years; Scheduled for primary unilateral TKA; Willing to participate and provide informed consent. Exclusion criteria: Required pain rescue medication ≥2 times within 24 h; Impaired Chinese language comprehension or communication; Chronic opioid use; Participation in other clinical trials during the study period; Development of severe acute complications during observation. Hertzog's (14) study pointed out that a cohort of 200 people can achieve more than 80% statistical efficiency at five time points. Considering a 20% loss to follow-up rate, the sample size should be no less than 240 cases.

### 2.2 Survey instruments

#### 2.2.1 General information questionnaire

The research team designed a general information questionnaire based on a review of previous literature. It included: age, gender, ethnicity, residence, body mass index (BMI), alcohol consumption history, smoking history, sleep quality, comorbidities, history of knee replacement, disease duration, Level of knee pain during preoperative activities, anesthesia method, use of patient-controlled analgesia, supplementary medication use, and surgical side.

#### 2.2.2 Numeric rating scale (NRS) for pain

The Numeric Rating Scale (NRS) used an 11-point scale (0–10), where patients rated their subjective pain intensity. Scores were categorized into four levels: 0 (no pain), 1–3 (mild pain), 4–6 (moderate pain), and 7–10 (severe pain).

#### 2.2.3 Pain catastrophizing scale (PCS)

The Pain Catastrophizing Scale (PCS) was developed by Sullivan et al. (15) and translated into Chinese by Yap et al. (16). The Chinese version includes three dimensions: helplessness (6 items), magnification (3 items), and rumination (4 items), totaling 13 items. It uses a 5-point Likert scale, with total scores ranging from 0 to 52. Higher scores indicate greater pain catastrophizing, with a score >30 indicating clinically significant pain catastrophizing. In this study, the Cronbach's α coefficient for PCS was 0.897.

#### 2.2.4 Hospital anxiety and depression scale (HADS)

The Hospital Anxiety and Depression Scale (HADS), developed by Zigmond et al. (17), consists of two dimensions (anxiety and depression) with 14 items total. Scores range from 0 to 21, with higher scores indicating more severe anxiety or depressive symptoms. A score ≥8 suggests the presence of anxiety or depression. In this study, the Cronbach's α coefficients for anxiety and depression were 0.756 and 0.760, respectively.

#### 2.2.5 Western Ontario and McMaster universities osteoarthritis index (WOMAC)

The WOMAC, developed by Bellamy et al. (18) and translated into Chinese by Xie et al. (19), includes three dimensions: pain (5 items), stiffness (2 items), and physical function (17 items), totaling 24 items. This study used a 5-point Likert scale (0–4), with total scores ranging from 0 to 96. Higher scores indicate more severe osteoarthritis symptoms. The overall Cronbach's α coefficient for WOMAC in this study was 0.89.

#### 2.2.6 Family care index questionnaire (FCIQ)

The Family Care Index Questionnaire (FCIQ), developed by Smilkstein et al. (20), consists of 5 items rated on a 3-point scale (0 = “rarely”, 1 = “sometimes”, 2 = “often”), with total scores ranging from 0 to 10. Higher scores indicate better family functioning. Scores are categorized as: 0–3 (severe family dysfunction), 4–6 (moderate dysfunction), and 7–10 (good family function). In this study, the Cronbach's α coefficient for FCIQ was 0.753.

### 2.3 Data collection and quality control

This study adopted a longitudinal multi-timepoint design with data collection at the following intervals: 1–2 days preoperatively (T0), postoperative day 1 (T1), day 2 (T2), day 3 (T3), day 5 (T4), and 3 months postoperatively (T5). Before survey administration, researchers explained the study purpose, significance, and questionnaire completion methods in detail to participants and obtained informed consent. Patients completed questionnaires independently, while for those unable to do so, researchers conducted face-to-face interviews and faithfully recorded responses. At T0, researchers administered paper-based versions of the general information questionnaire, NRS, PCS, HADS, and APGAR questionnaire through face-to-face interviews in orthopedic wards. For postoperative assessments at T1, T2, T3, and T4, patients' self-reported pain levels during activity (daily postoperative exercises, walking, and flexion/extension movements assisted by a rehabilitation physician) were collected using NRS at 5 PM each day through face-to-face interviews. At the 3-month postoperative follow-up (T5), patients' joint functional recovery was assessed via telephone using the WOMAC scale. To ensure data accuracy and reliability, this study implemented rigorous quality control measures. First, all collected data were processed and entered by two independent researchers. Second, all patients received standardised basic analgesia. When breakthrough pain persisted for 30 min (21), a rescue dose of 50 mg of buccinnazine hydrochloride was administered via intramuscular injection. The basic analgesia protocol included preoperative pain management education and intravenous infusion of cyclooxygenase-2 (COX-2) inhibitors for prophylactic analgesia; intraoperative periarticular “cocktail” injection with a formulation of ropivacaine, epinephrine, ketorolac, and morphine, diluted with normal saline to a total volume of 40 ml; postoperative intravenous infusion of nonsteroidal anti-inflammatory drugs and oral tramadol tablets; and concurrent use of ice packs, ear acupuncture, Chinese herbal poultices, and moxibustion for traditional Chinese medicine analgesia. Third, to best capture the natural progression of postoperative pain, pain scores were recorded immediately before any rescue medication administration. Fourth, the Patient-Controlled Analgesia (PCA) weaning protocol: On the morning of the first postoperative day, when the patient's pain is stably controlled (NRS rest score consistently ≤4), without severe side effects, and once mobilization has begun, the process is initiated. First, discontinue the background infusion of the PCA pump while retaining the PCA bolus function for rescue use, and simultaneously initiate regular oral administration of tramadol. Monitor the patient's frequency of rescue oral medication requests and pain scores. If over the next 4–6 h, the patient's pain remains well-controlled without frequent use of PCA bolus (e.g., usage frequency <2 times/4 h), completely discontinue the PCA. If pain becomes uncontrolled (NRS ≥7) after discontinuation, restart the PCA background infusion and reassess after 4 h. The study did not interfere with clinical analgesic decisions, prioritizing patients' pain management needs throughout. This study was approved by the hospital ethics committee (Approval No. 2024-10-005).

### 2.4 Statistical analysis

Data analysis was performed using SPSS 27.0 and Mplus 8.3 software. Categorical variables were described using frequencies and percentages, with between-group comparisons conducted using chi-square tests or Fisher's exact test. Measurement data following a normal distribution are described using mean ± standard deviation. Intergroup comparisons were performed using the independent samples *t*-test and one-way analysis of variance (ANOVA). Non-normally distributed continuous data were described using medians and interquartile ranges, with between-group comparisons performed using the Mann–Whitney *U*-test and the Kruskal–Wallis *H*-test. Latent growth curve modeling was employed to characterize the overall developmental trajectory of APSP in patients undergoing TKA. GMM was employed to examine the changing trajectories of APSP in TKA patients across T1-T4 time points and to identify potential heterogeneous subgroups. Logistic regression analysis was used to assess the influence of relevant variables on APSP, while multiple linear regression was applied to investigate the relationship between trajectory patterns and joint functional recovery at 3 months postoperatively. A *p*-value <0.05 was considered statistically significant.

## 3 Results

### 3.1 Baseline characteristics and scale scores

**Figure 1:**
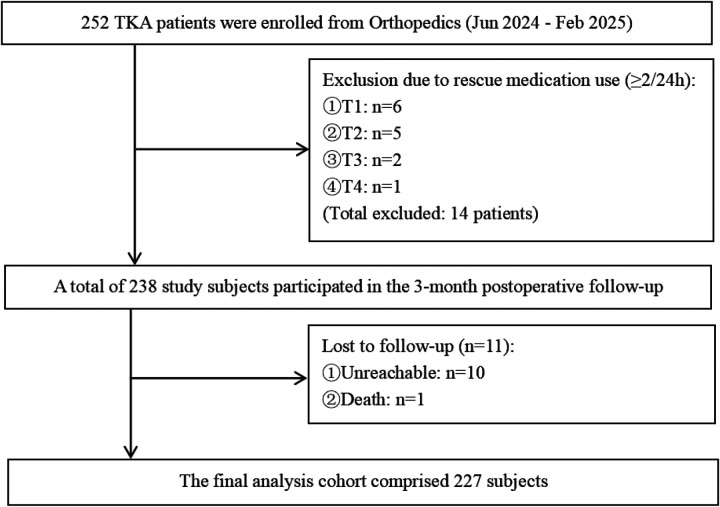
Flowchart of Patient Recruitment and Follow-up.

A total of 252 questionnaires were distributed. Among these, 11 cases were lost to follow-up, and 14 cases required rescue medication≥2 times within 24 h, resulting in 227 valid questionnaires retrieved. The effective response rate was 89.72%, as detailed in Figure 1. The general characteristics and scale scores of the surveyed participants are presented in Table 1. A comparison of baseline characteristics between the excluded and included groups showed no statistically significant differences in any indicators, with observed effect sizes being minimal. This indicates that the exclusion process did not introduce significant selection bias, and the final sample included for analysis demonstrated good representativeness at baseline, thereby supporting the internal validity of subsequent findings. Details are provided in Supplementary Table S1. Since the proportion of participants lost to follow-up was less than 5%, only descriptive statistics of their baseline information are presented in Supplementary Table S2.

**Table 1:** Univariate analysis of demographic characteristics by acute post-TKA pain trajectory subgroups (*n* = 227).

Variable	Total sample (*n* = 227)	Moderate-high persistent pain group (*n* = 101)	Moderate-low rapid relief group (*n* = 126)	χ*^2/^/t/Z*	*p*-value
Age [years, M (P25, P75)]	66.28 (60, 72)	69 (62, 74)	64 (58, 71)	−3.658**	<0.001
Gender [*n* (%)]					
Male	47 (20.7)	13 (27.66)	34 (72.34)	6.801*	0.009
Female	180 (79.3)	88 (48.89)	92 (51.11)		
Ethnicity [*n* (%)]					
Other	43 (18.94)	18 (41.86)	25 (58.14)	0.149*	0.7
Han	184 (81.06)	83 (45.11)	101 (54.89)		
Residence [*n* (%)]					
Rural	144 (63.44)	65 (45.14)	79 (54.86)	0.066*	0.797
Urban	83 (36.56)	36 (43.37)	47 (56.63)		
BMI [kg/m², M (P25, P75)]	24.95 (22.35, 27.68)	25 (22.36, 28.43)	24.56 (22.28, 27.08)	−1.194**	0.233
Smoking history [*n* (%)]					
No	194 (85.46)	91 (46.91)	103 (53.09)	3.148*	0.76
Yes	33 (14.54)	10 (30.3)	23 (69.7)		
Alcohol use [*n* (%)]					
No	165 (72.69)	74 (44.85)	91 (55.15)	0.031*	0.861
Yes	62 (27.31)	27 (43.55)	35 (56.45)		
Sleep quality [*n* (%)]					
Poor	100 (44.05)	63 (63)	37 (37)	24.788*	<0.001
Good	127 (55.95)	38 (29.92)	89 (70.08)		
Comorbidities [*n* (%)]					
None	75 (33.04)	18 (24)	57 (76)	19.047*	<0.001
Present	152 (66.96)	83 (54.61)	69 (45.39)		
Prior knee surgery [*n* (%)]					
No	182 (80.18)	88 (48.35)	94 (51.65)	5.534*	0.019
Yes	45 (19.82)	13 (28.89)	32 (71.11)		
Disease duration [*n* (%)]					
<5years	71 (31.28)	36 (50.7)	35 (49.3)	1.821*	0.402
5–10 years	122 (53.74)	52 (42.62)	70 (57.38)		
>10 years	34 (14.98)	13 (38.24)	21 (61.76)		
Preoperative pain [M (P25, P75)]	6.18 (6, 6)	7 (6, 7)	6 (5, 6)	−8.457**	<0.001
Anesthesia type [*n* (%)]					
Combined spinal-epidural anesthesia	111 (48.9)	45 (40.54)	66 (59.46)	1.374*	0.241
General anesthesia	116 (51.1)	56 (48.28)	60 (51.72)		
Patient-controlled analgesia pump [*n* (%)]					
No	40 (17.62)	21 (52.5)	19 (47.5)	1.26*	0.262
Yes	187 (82.38)	80 (42.78)	107 (57.22)		
Adjunctive meds [*n* (%)]					
No	149 (65.64)	70 (46.98)	79 (53.02)	1.086*	0.297
Yes	78 (34.36)	31 (39.74)	47 (60.26)		
Surgical side [*n* (%)]					
Left	102 (44.93)	41 (40.2)	61 (59.8)	1.385*	0.239
Right	125 (55.07)	60 (48)	65 (52)		
PCS score [M (P25, P75)]	30.27 (24, 37)	36 (33.5, 38)	26 (22, 32.25)	−7.858**	<0.001
FCIQ score [M (P25, P75)]	6.73 (5, 8)	6 (5, 7)	7 (6, 8)	−4.418**	<0.001
HADS-anxiety [M (P25, P75)]	5.33 (3, 7)	7 (4, 8)	4 (3, 6)	−6.006**	<0.001
HADS-depression [M (P25, P75)]	6.78 (5, 8)	8 (6, 10)	6 (5, 7)	−6.324**	<0.001
WOMAC score [mean ± SD]	30.908 ± 6.392	35.406 ± 5.138	27.302 ± 4.827	12.131***	<0.001

Data formats: Values are displayed as *N* (%) for frequencies and percentages, or M (P25, P75) for medians and interquartile ranges.

* χ^2^ value.

** *Z*-score.

*** *t*-value.

### 3.2 Analysis of acute postoperative pain trajectories

**Figure 2:**
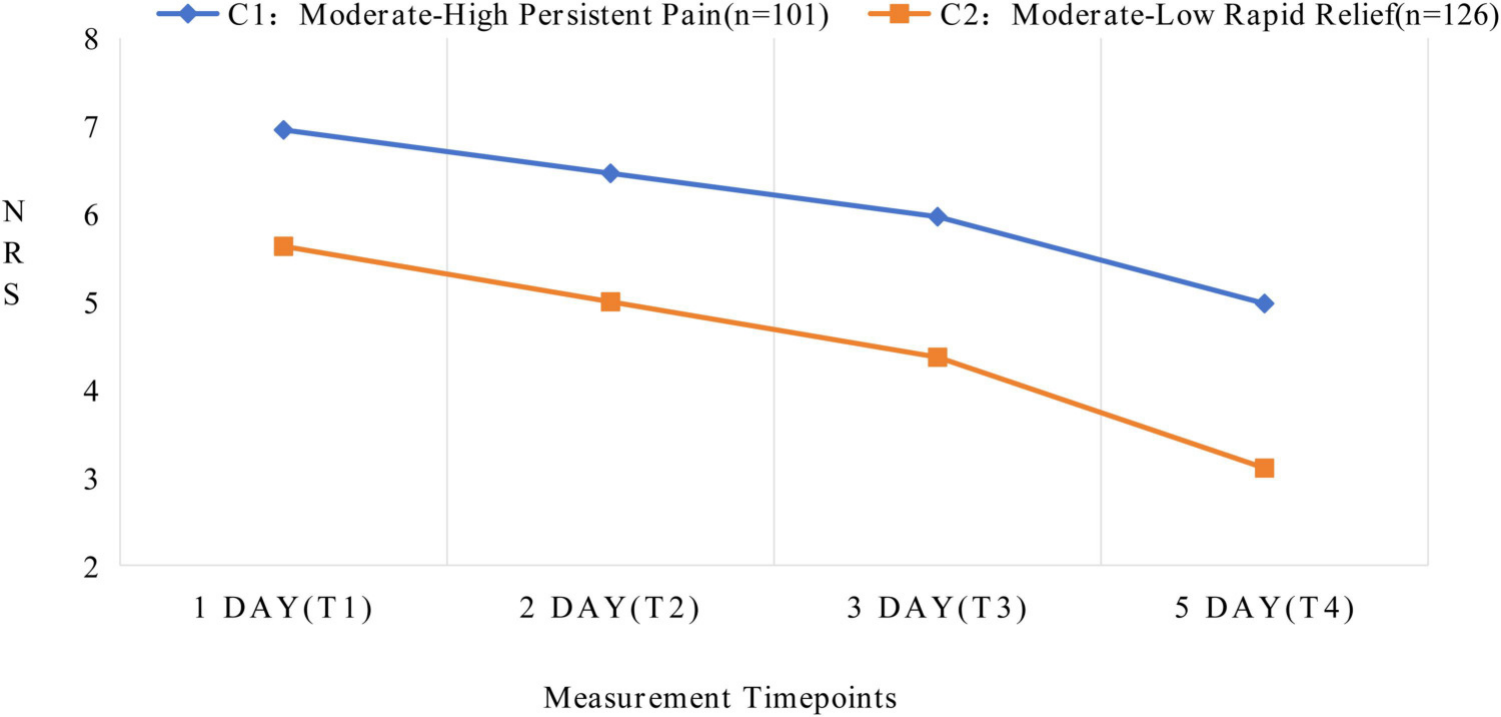
Developmental trajectories of latent classes for acute postoperative pain following total knee arthroplasty.

Latent growth curve modeling was employed to fit both linear and quadratic (nonlinear) models. Based on model fit indices, the linear model was ultimately selected for further analysis, as detailed in Table 2. GMM was applied by incrementally increasing the number of classes from 1 to 3. No covariates were included in any of the models. As the number of classes increased, the values of Information Criterion (AIC), Bayesian Information Criterion (BIC), and Adjusted Bayesian Information Criterion (ABIC) progressively decreased. When the number of classes was set to three, the Lo-Mendell-Rubin (LMR) test did not reach statistical significance (*P* = 0.831). Moreover, the smallest trajectory subgroup accounted for only 5.7% of the total sample, comprising a relatively small number of individuals. This subgroup demonstrated low clinical interpretability and lacked credibility for generalization. Therefore, after comprehensive consideration of clinical utility and the above model fit indices, the two-class linear GMM was ultimately retained as the optimal model. Detailed fit indices are presented in Table 3. The average posterior probabilities for class membership were 0.980 and 0.972 for each class, respectively. The mean pain score distributions across postoperative time points for each class are shown in Figure 2.

**Table 2:** Parameter estimates of the latent growth curve model for acute pain in patients undergoing total knee arthroplasty (*n* = 227).

Model	χ^2^	*df*	P	CFI	TLI	SRMR	RMSEA
Nonlinear	239.111	5	<0.001	0.77	0.724	0.208	0.454
Linear	3.8	1	0.0513	0.997	0.983	0.016	0.111

CFI, comparative fit index; TLI, Tucker–Lewis index; SRMR, standardized root mean square residual; RMSEA, root mean square error of approximation. Generally, CFI/TLI >0.90, SRMR <0.08, and RMSEA <0.08 indicate acceptable model fit.

**Table 3:** Fit indices of the growth mixture models for acute postoperative pain following total knee arthroplasty (*n* = 227).

Model	AIC	BIC	ABIC	Entropy	LMR	BLRT	Class Probabilities
1	1,722.973	1,753.797	1,725.274	–	–	–	1
2	1,651.521	1,702.895	1,655.356	0.881	0.009	<0.001	0.452/0.548
3	1,213.321	1,264.696	1,217.156	1	0.831	<0.001	0.0573/0.621/0.322

AIC, Akaike information criterion; BIC, Bayesian information criterion; ABIC, adjusted Bayesian information criterion; lower values indicate better model fit. Entropy is a measure of classification accuracy, ranging from 0 to 1, with higher values (typically >0.60) indicating better distinction between classes. BLRT, bootstrap likelihood ratio test; a significant *p*-value (<0.05) supports that the model with k classes fits better than the model with k-1 classes. Class Probabilities represent the average latent class probabilities for most likely class membership.

The two distinct latent class trajectories of postoperative pain in TKA patients demonstrated different characteristics at each time point. Based on the changing patterns and features of pain trajectories, each latent class was named accordingly. See Supplementary Table S3 for details. In Class 1 (C1), patients exhibited higher initial pain levels (intercept = 6.956, *P* < 0.001) with a relatively slower decline over time (slope = −0.494, *P* < 0.001), maintaining moderate pain levels even on postoperative day 5. Therefore, C1 was designated as the “Moderate-High Persistent Pain” group. In Class 2 (C2), patients showed lower initial pain levels (intercept = 5.631, *P* < 0.001) with a steeper declining trend (slope = −0.631, *P* < 0.001), demonstrating significant pain relief by postoperative day 5. Consequently, C2 was named the “Moderate-Low Rapid Relief” group.

### 3.3 Univariate analysis of acute postoperative pain trajectories

The results demonstrated statistically significant differences (*P* < 0.05) in APSP trajectory development among TKA patients based on age, gender, sleep quality, comorbidities, history of knee replacement, preoperative NRS scores, PCS scores, FCIQ scores, and anxiety/depression levels (Table 1).

### 3.4 Multivariate analysis of acute postoperative pain trajectories

Using APSP trajectory categories as the dependent variable (with “Moderate-Low Rapid Relief” as reference) and incorporating all univariate predictors with *P* < 0.05, logistic regression analysis identified age, preoperative NRS, PCS, and FCIQ scores as significant independent predictors of APSP trajectories (*P* < 0.05, Table 4).

**Table 4:** Binary logistic regression analysis of potential class membership in acute post-TKA pain trajectories (*n* = 227).

Variable	*β* coefficient	*SE*	*Wald* χ*^2^*	*p*-value	*OR*	95% CI
Constant	−13.526	2.854	22.453	<0.001	–	–
Age	0.05	0.024	4.575	0.032	1.052	1.004–1.101
Preoperative pain	1.266	0.341	13.804	<0.001	3.546	1.819–6.914
PCS score	0.088	0.034	6.839	0.009	1.092	1.022–1.167
FCIQ score	−0.27	0.122	4.87	0.027	0.763	0.601–0.97

The independent variables (age, preoperative pain level, pain catastrophizing, and family care index) were entered as raw values. The dependent variable was coded as: Moderate-Low Rapid Relief group=0, Moderate-High Persistent Pain group = 1.

### 3.5 Impact of acute postoperative pain trajectories on 3-month joint functional recovery

After controlling for age, preoperative NRS, PCS, and FCIQ scores, multiple linear regression analysis was performed with APSP trajectory categories as the independent variable and WOMAC scores as the dependent variable. Dummy variable coding was applied, using the “Moderate-Low Rapid Relief” group as the reference (coded as 0), while the “Moderate-High Persistent Pain” group was coded as 1. As shown in Table 5, compared to TKA patients in the “Moderate-Low Rapid Relief” subgroup, those in the “Moderate-High Persistent Pain” subgroup were associated with significantly worse functional outcomes (*β* = 0.32, *t* = 5.64, *p* < 0.001).

**Table 5:** Results of multilevel linear regression analysis on the association between acute post-TKA pain trajectories and 3-month postoperative knee function recovery (*n* = 227).

Variable	Model 1				Model 2			
	*β*	*SE*	*t*	*P*	*β*	*SE*	*t*	*P*
Age	0.288	0.037	5.922	<0.001	0.248	0.035	5.379	<0.001
Preoperative pain	0.276	0.487	4.465	<0.001	0.179	0.476	2.95	0.004
PCS score	0.303	0.052	5.068	<0.001	0.207	0.051	3.54	<0.001
FCIQ score	−0.181	0.21	−3.699	<0.001	−0.138	0.199	−2.977	0.003
Acute pain trajectories (with “Moderate-low rapid relief” as reference)	–	–	–	–	0.32	0.729	5.64	<0.001
*R^2^*	0.508				0.57			
Δ*R^2^*	0.508 (compared to null model)				0.062 (incremental to Model 1)			
*F*	57.356				58.614			
*P*	<0.001				<0.001			

## 4 Discussion

### 4.1 Distinct acute pain trajectories exist after total knee arthroplasty

Using GMM, this study identified two latent classes of APSP trajectories in TKA patients: the moderate-high persistent pain group (45.2%) and the moderate-low rapid relief group (54.8%), demonstrating the heterogeneous nature of postoperative pain, Similar to the conclusions of Thomazeau (10). Furthermore, Thomazeau found at the 6-month postoperative follow-up that the high pain intensity group had a significantly higher incidence of chronic pain compared to the low pain intensity group (10). Therefore, healthcare providers need to early identify patients with high pain scores and low rates of pain relief, promptly adjust intervention strategies, and implement stepped, personalized treatment measures. Rehabilitation therapists should adopt differentiated rehabilitation interventions based on distinct pain trajectories. The moderate-high persistent pain group exhibited significant prolonged postoperative pain characteristics, with activity-related NRS scores remaining at relatively high levels during the first 5 postoperative days. This may be associated with preoperative central sensitization (22), health status (7), and psychological factors (23). Therefore, for this subgroup, comprehensive management strategies should be implemented, including enhanced multimodal analgesia, psychological interventions, modified rehabilitation protocols, and surgical optimization to prevent pain chronification. Patients in the “moderate-low rapid relief group” subgroup exhibited a rapid decline in postoperative activity-related pain, suggesting a favorable response to standard multimodal analgesia. This subgroup may derive greater benefit from ERAS protocols, thereby optimizing functional outcomes. The underlying neurophysiological and psychological mechanisms warrant further investigation.

### 4.2 Analysis of influencing factors for acute postoperative pain trajectories following total knee arthroplasty

#### 4.2.1 Age

The results of this study demonstrate that compared to the moderate-low rapid relief group, older adult patients are more likely to develop moderate-high persistent pain patterns, a finding consistent with the research conclusions of Chen et al. (24). This age-related difference in pain trajectories may be associated with pre-existing central sensitization and slowed opioid metabolism, among other factors (25). Morze (8) conducted a prospective cohort study observing weekly dynamic changes in pain among TKA patients over three postoperative months, confirming that older adult TKA patients exhibit significant delays in pain recovery. Combined with our findings, these results indicate that age influences both the acute-phase occurrence and long-term resolution of post-TKA pain through various mechanisms. Based on these conclusions, we recommend establishing specialized follow-up protocols for older adult patients in clinical practice, implementing early pain assessment and intervention strategies to reduce the risk of adverse outcomes.

#### 4.2.2 Preoperative pain and pain catastrophizing

The study demonstrated that patients with higher preoperative NRS and PCS scores were more likely to develop the moderate-high persistent pain pattern, aligning with findings from Stessel (26) and Giordano (27). Research indicates that patients with higher levels of preoperative pain exhibit increased neuronal sensitivity to nociceptive signals and sensitization of the peripheral or central nervous system, leading to hyperalgesia and consequently enhancing the intensity and duration of pain perception (28). Concurrently, pain catastrophizing reinforces attentional bias, leading to central sensitization and pain memory consolidation, collectively amplifying postoperative pain perception (28). Therefore, we recommend incorporating NRS and PCS into routine preoperative assessments for TKA patients. For high-risk patients, standardized pharmacological therapy should be combined with non-pharmacological interventions such as Cognitive Behavioral Therapy (CBT) to optimize pain management outcomes.

#### 4.2.3 Family support level

The results of this study show that TKA patients with lower levels of family support were more likely to develop the moderate-high persistent pain pattern, indicating that good family support has significant protective effects. This protective effect is primarily achieved through the social support buffering theory (29): at the physiological level, it can effectively reduce stress response intensity and inflammatory reactions (30); at the behavioral level, it can improve treatment compliance and promote standardized medication use (31); at the psychological level, it can alleviate pain-related negative cognition and enhance confidence in pain coping (32). A study on hip replacement patients found that negative social support (such as excessive stress or criticism from significant family members) may have a more significant association with pain relief and functional recovery than positive support (33). This finding suggests that future research could further focus on the impact mechanisms of negative social support on postoperative recovery, in order to provide more targeted strategies for clinical interventions.

### 4.3 Impact of acute postoperative pain trajectories on 3-month joint functional recovery following total knee arthroplasty

The results demonstrated that the APSP trajectory served as an independent predictor. Compared to patients in the moderate-low rapid relief group, those in the moderate-high persistent pain group exhibited significantly worse WOMAC scores, with this variable alone increasing the model's explained variance by 6.2%. Notably, it is predictive potency (*β* = 0.32) even exceeded that of variables such as age, preoperative pain level, and pain catastrophizing. These findings indicate that patients experiencing severe acute movement-related pain face substantially elevated risks of poor functional recovery, corroborating previous studies by Singh (9) and Lo (34). The underlying mechanism may involve kinesiophobia induced by intense pain, which significantly reduces patients' willingness and frequency to participate in early rehabilitation exercises, thereby delaying functional recovery (35). Contemporary research in pain medicine has demonstrated that pain is an active process resulting from the interplay of physiological and psychological factors, and that pain perception can be effectively modulated through psychological interventions (36). Therefore, healthcare providers can utilize psychological approaches such as preoperative health education, cognitive-behavioral therapy, mindfulness training, and pain empathy to enhance patients' emotional regulation, alleviate postoperative pain, and improve self-management capabilities. Notably, during the follow-up period, we observed that some patients with high early pain scores showed significant improvement in activity-related pain at 3 months postoperatively, yet demonstrated limited improvement in joint function. This “pain-function recovery dissociation” suggests that pain relief and functional recovery may be mediated by distinct pathophysiological mechanisms. Further investigation into the underlying influencing factors is warranted, as traditional pain-oriented postoperative management strategies may be insufficient to ensure optimal functional outcomes. Future studies should establish a dual-track evaluation system integrating both pain and functional recovery to further elucidate the relationship between these two domains.

## 5 Conclusions

This study identified two distinct acute postoperative pain trajectories in TKA patients using GMM, with each trajectory demonstrating unique characteristics. The trajectories were significantly influenced by age, preoperative pain levels, pain catastrophizing, and family support. Particular clinical attention should be given to patients exhibiting the moderate-to-high persistent pain pattern, with individualized multimodal analgesia and rehabilitation strategies tailored to each trajectory's specific characteristics. Several limitations warrant consideration. First, the single-center design may limit generalizability, necessitating future multicenter studies with larger sample sizes. Second, the 3-month postoperative follow-up period requires extension to evaluate long-term pain and functional outcomes. Third, the assessment of pain in this study relied solely on patients' subjective reports. Future research could incorporate objective evaluation tools—such as electromyography, galvanic skin response, and computer vision-based analysis of facial micro-expressions—to enable high-frequency longitudinal observations and facilitate an in-depth analysis of the dynamic patterns underlying pain progression. Fourth, the conclusions of this study apply to patients who respond to basic analgesic regimens and should not be generalized to refractory pain subgroups requiring frequent rescue analgesia. Finally, the exclusive focus on movement-induced pain during the acute phase underscores the importance of future research examining both resting and activity-related pain trajectories to better understand their dynamic interplay and optimize rehabilitation protocol matching.

## Data Availability

The raw data supporting the conclusions of this article will be made available by the authors, without undue reservation.

## References

[1] McGrory BJ Weber KL Jevsevar DS Sevarino K. Surgical management of osteoarthritis of the knee: evidence-based guideline. J Am Acad Orthop Surg. (2016) 24(8):e87–93. 10.5435/JAAOS-D-16-00159 27355286

[2] Chen LQ Wu B Hong AM Xu CF Zai ZX Li J. Survey on acute postoperative pain status. J Clin Anesthesiol. (2021) 37(11):1200–3. 10.12089/jca.2021.11.017

[3] Tang H Du H Tang Q Yang D Shao H Zhou Y. Chinese Patients’ satisfaction with total hip arthroplasty: what is important and dissatisfactory? J Arthroplasty. (2014) 29(12):2245–50. 10.1016/j.arth.2013.12.032 24524778

[4] Xu JG. Expert consensus on postoperative pain management in adults. J Clin Anesthesiol. (2017) 33(09):911–7. Available online at: https://kns.cnki.net/kcms2/article/abstract?v=AA8hwJ51-CSzub1uz33F0V8C1CCOlzqt96U-hmGl1P4ZOhnUmKRHpxgcOpAWr-xkuueUkvAQRFfABlWjJBfYfJKay9EmkekoxtFwgTUAZoI63v6yCzmr8pDE6lssQWYh5rblUOkJleeAM6mPDzVOpccC5iprncUU_vX8yPkqaKGV2KXcxUJYAGGR6Iq9VRIcsOrdnVPJvyg=&uniplatform=NZKPT&language=CHS

[5] Puolakka PA Rorarius MG Roviola M Puolakka TJ Nordhausen K Lindgren L. Persistent pain following knee arthroplasty. Eur J Anaesthesiol. (2010) 27:455–60. 10.1097/EJA.0b013e328335b31c 20299989

[6] Riddle DL Dumenci L. Patient acceptable symptom state versus latent class analysis outcome classification: a comparative longitudinal study of knee arthroplasty. Arthritis Care Res (Hoboken). (2023) 75(7):1519–26. 10.1002/acr.24962 35638702 PMC9708946

[7] Ellyson AM Gordon G Zhou C Rabbitts JA. Trajectories, risk factors, and impact of persistent pain after major musculoskeletal surgery in adolescents: a replication study. J Pain. (2022) 23(6):995–1005. 10.1016/j.jpain.2021.12.009 34974171 PMC9232895

[8] Morze CJ Johnson NR Williams G Moroney M Lamberton T McAuliffe M. Knee pain during the first three months after unilateral total knee arthroplasty: a multi-centre prospective cohort study. J Arthroplasty. (2013) 28(9):1565–70. 10.1016/j.arth.2013.02.029 23541867

[9] Singh JA Lemay CA Nobel L Yang W Weissman N Saag KG Association of early postoperative pain trajectories with longer-term pain outcome after primary total knee arthroplasty. JAMA Netw Open. (2019) 2(11):e1915105. 10.1001/jamanetworkopen.2019.15105 31722026 PMC6902788

[10] Thomazeau J Rouquette A Martinez V Rabuel C Prince N Laplanche JL Predictive factors of chronic post-surgical pain at 6 months following knee replacement: influence of postoperative pain trajectory and genetics. Pain Physician. (2016) 19(5):E729–41. 27389116

[11] Althaus A Arránz Becker O Moser KH Lux EA Weber F Neugebauer E Postoperative pain trajectories and pain chronification—an empirical typology of pain patients. Pain Med. (2018) 19(12):2536–45. 10.1093/pm/pny099 29800281

[12] Awadalla SS Winslow V Avidan MS Haroutounian S Kannampallil TG. Effect of acute postsurgical pain trajectories on 30-day and 1-year pain. PLoS One. (2022) 17(6):e0269455. 10.1371/journal.pone.0269455 35687544 PMC9187125

[13] Lavand'homme PM Grosu I France MN Thienpont E. Pain trajectories identify patients at risk of persistent pain after knee arthroplasty: an observational study. Clin Orthop Relat Res. (2014) 472(5):1409–15. 10.1007/s11999-013-3389-5 24258688 PMC3971216

[14] Hertzog C Lindenberger U Ghisletta P Oertzen Tv. On the power of multivariate latent growth curve models to detect correlated change. Psychol Methods. (2006) 11:244–52. 10.1037/1082-989X.11.3.244 16953703

[15] Sullivan MJL Bishop SR Pivik J. The pain cata-strophizing scale: development and validation. Psychol Assess. (1995) 7(4):524–32. 10.1037/1040-3590.7.4.524

[16] Yap JC Lau J Chen PP Gin T Wong T Chan I Validation of the Chinese pain catastrophizing scale (HK-PCS) in patients with chronic pain. Pain Med. (2008) 9(2):186–95. 10.1111/j.1526-4637.2007.00307.x 18298701

[17] Zigmond AS Snaith RP. The hospital anxiety and depression scale. Acta Psychiatr Scand. (1983) 67(6):361. 10.1111/j.1600-0447.1983.tb09716.x 6880820

[18] Bellamy N Buchanan WW Goldsmith CH Campbell J Stitt LW. Validation study of WOMAC: a health status instrument for measuring clinically important patient relevant outcomes to antirheumatic drug therapy in patients with osteoarthritis of the hip or knee. J Rheumatol. (1988) 15(12):1833–40. 3068365

[19] Xie F Li SC Goeree R Tarride JE O'Reilly D Lo NN Validation of Chinese western Ontario and McMaster universities osteoarthritis index (WOMAC) in patients scheduled for total knee replacement. Qual Life Res. (2008) 17(4):595–601. 10.1007/s11136-008-9340-7 18415706

[20] Smilkstein G. The family APGAR: a proposal for a family function test and its use by physicians. J Fam Pract. (1978) 6(6):1231–9. 660126

[21] Dworkin RH Turk DC Farrar JT Haythornthwaite JA Jensen MP Katz NP Core outcome measures for chronic pain clinical trials: IMMPACT recommendations. Pain. (2005) 113(1-2):9–19. 10.1016/j.pain.2004.09.012 15621359

[22] Kim SH Yoon KB Yoon DM Yoo JH Ahn KR. Influence of centrally mediated symptoms on postoperative pain in osteoarthritis patients undergoing total knee arthroplasty: a prospective observational evaluation. Pain Pract. (2015) 15(6):E46–53. 10.1111/papr.12311 25980527

[23] Fernández-de-Las-Peñas C Florencio LL de-la-Llave-Rincón AI Ortega-Santiago R Cigarán-Méndez M Fuensalida-Novo S Prognostic factors for postoperative chronic pain after knee or hip replacement in patients with knee or hip osteoarthritis: an Umbrella review. J Clin Med. (2023) 12(20):6624. 10.3390/jcm12206624 37892762 PMC10607727

[24] Chen DX Zhang YY Liu J Chen Y. Postoperative acute pain trajectory and chronic postsurgical pain after abdominal surgery: a prospective cohort study and mediation analysis. Int J Surg. (2025) 111(2):1968–76. 10.1097/JS9.0000000000002218 39903529

[25] Mercadante S Casuccio A Pumo S Fulfaro F. Factors influencing the opioid response in advanced cancer patients with pain followed at home: the effects of age and gender. Support Care Cancer. (2000) 8(2):123–30. 10.1007/s005200050026 10739359

[26] Stessel B Fiddelers AA Marcus MA van Kuijk SM Joosten EA Peters ML External validation and modification of a predictive model for acute postsurgical pain at home after day surgery. Clin J Pain. (2017) 33(5):405–13. 10.1097/AJP.0000000000000413 27428546 PMC5638419

[27] Giordano NA Kent ML Kroma RB Rojas W Lindl MJ Lujan E Acute postoperative pain impact trajectories and factors contributing to trajectory membership. Pain Med. (2023) 24(7):829–36. 10.1093/pm/pnac203 36579887

[28] Gerum M Simonin F. Behavioral characterization, potential clinical relevance and mechanisms of latent pain sensitization. Pharmacol Ther. (2022) 233:108032. 10.1016/j.pharmthera.2021.108032 34763010

[29] Cohen S Wills TA. Stress, social support, and the buffering hypothesis. Psychol Bull. (1985) 98(2):310–57. 10.1037/0033-2909.98.2.310 3901065

[30] Uchino BN. Social support and health: a review of physiological processes potentially underlying links to disease outcomes. J Behav Med. (2006) 29(4):377–87. 10.1007/s10865-006-9056-5 16758315

[31] Guan MM Li WF Xu WW Ru L. Correlation analysis of postoperative self-efficacy, family support and rehabilitation exercise compliance in patients with spinal fracture. J Cervicodynia Lumbodynia. (2024) 45(06):1133–8. 10.3969/i.issn.1005-7234.2024.06.028

[32] Jiang R Yan J Wang Z Gao W Li RL. Influence of family participatory hospice care on pain mediator levels, self-perceived burden, and quality of life in patients with lung cancer. Chin Nurs Res. (2025) 39(07):1167–72. 10.12102/i.issn.1009-6493.2025.07.018

[33] Stephens MA Druley JA Zautra AJ. Older adults’ recovery from surgery for osteoarthritis of the knee: psychosocial resources and constraints as predictors of outcomes. Health Psychol. (2002) 21(4):377–83. 10.1037/0278-6133.21.4.377 12090680

[34] Lo LWT Suh J Chen JY Liow MHL Allen JC Lo NN Early postoperative pain after total knee arthroplasty is associated with subsequent poorer functional outcomes and lower satisfaction. J Arthroplasty. (2021) 36(7):2466–72. 10.1016/j.arth.2021.02.044 33744080

[35] Gao C Shi GH Liu JD Han X Gao B. Analysis of the current situation and influencing factors of agoraphobia in patients after total knee arthroplasty. Trauma Crit Care Med. (2025) 13(04):284–8. 10.16048/j.issn.2095-5561.2025.04.09

[36] Colvin LA Rice ASC. Progress in pain medicine: where are we now? Br J Anaesth. (2019) 123(2):e173–6. 10.1016/j.bja.2019.04.051 31174848 PMC6676231

