# Supplementary material for: Acute postoperative pain trajectories and their impact on functional recovery following total knee arthroplasty
Source: Front Pain Res (Lausanne). 2025 Oct 13;6:1659917. doi: 10.3389/fpain.2025.1659917 (PMC12554665; doi:10.3389/fpain.2025.1659917)
Supplement: Supplementary file 1 [file Table1.docx]

**Supplementary Material**

**Content**

Table S1. Comparison of Baseline Characteristics Between Analyzed and Excluded Patients ............... 2

Table S2. Baseline Characteristics of Analysed, Excluded, and Lost-to-Follow-up Patients ………….. 4

Table S3. Mean (Standard Error) of Intercept and Slope for Each Latent Class ………………….…… 6

Table S1. Comparison of Baseline Characteristics Between Analyzed and Excluded Patients

| **Variable** | **Analysed(n=227)** | **Excluded(n=14)** | **p-value** | **Effect Size** |
| --- | --- | --- | --- | --- |
| Age [years, M(P25,P75)] | 66.28(60,72) | 66.5(62.25,72.25) | 0.678 | 0.066* |
| Gender [n(%)] |  |  | 1 |  |
| Male | 47(20.7) | 3(21.43) |  | 0.73%† |
| Female | 180(79.3) | 11(78.57) |  |  |
| Ethnicity [n(%)] |  |  | 1 |  |
| Other | 43(18.94) | 2(14.29) |  | 4.65%† |
| Han | 184(81.06) | 12(85.71) |  |  |
| Residence [n(%)] |  |  | 0.949 |  |
| Rural | 144(63.44) | 9(64.29) |  | 0.85%† |
| Urban | 83(36.56) | 5(35.71) |  |  |
| BMI [kg/m², M(P25,P75)] | 24.95(22.35, 27.68) | 24.6(22.23,26.67) | 0.840 | 0.032* |
| Smoking History [n(%)] |  |  | 1 |  |
| No | 194(85.46) | 12(85.71) |  | 0.25%† |
| Yes | 33(14.54) | 2(14.29) |  |  |
| Alcohol Use [n(%)] |  |  | 1 |  |
| No | 165(72.69) | 10(71.43) |  | 1.26%† |
| Yes | 62(27.31) | 4(28.57) |  |  |
| Sleep Quality [n(%)] |  |  | 0.93 |  |
| Poor | 100(44.05) | 6(42.86) |  | 1.19%† |
| Good | 127(55.95) | 8(57.14) |  |  |
| Comorbidities [n(%)] |  |  | 1 |  |
| None | 75(33.04) | 5(35.71) |  | 2.67%† |
| Present | 152(66.96) | 9(64.29) |  |  |
| Prior Knee Surgery [n(%)] |  |  | 1 |  |
| No | 182(80.18) | 11(78.57) |  | 1.61%† |
| Yes | 45(19.82) | 3(21.43) |  |  |
| Disease Duration [n(%)] |  |  | 0.941 |  |
| ＜5years | 71(31.28) | 5(35.71) |  | 4.43%† |
| 5-10 years | 122(53.74) | 7(50.00) |  |  |
| ＞10 years | 34(14.98) | 2(14.29) |  |  |
| Preoperative Pain [M(P25,P75)] | 6.18(6,6) | 6(6,6) | 0.908 | 0.017* |
| Anesthesia Type [n(%)] |  |  | 0.936 |  |
| Combined Spinal-Epidural Anesthesia | 111(48.9) | 7(50) |  | 1.1%† |
| General Anesthesia | 116(51.1) | 7(50) |  |  |
| Patient-Controlled Analgesia Pump [n(%)] |  |  | 1 |  |
| No | 40(17.62) | 2(14.29) |  | 3.33%† |
| Yes | 187(82.38) | 12(85.71) |  |  |
| Adjunctive Meds [n(%)] |  |  | 1 |  |
| No | 149（65.64） | 9（64.29） |  | 1.35%† |
| Yes | 78（34.36） | 5（35.71） |  |  |
| Surgical Side [n(%)] |  |  | 0.879 |  |
| Left | 102(44.93) | 6(42.86) |  | 2.07%† |
| Right | 125(55.07) | 8(57.14) |  |  |
| PCS Score [M(P25,P75)] | 30.27(24,37) | 30.5(23.5,36.25) | 0.796 | 0.041* |
| FCIQ Score [M(P25,P75)] | 6.73(5,8) | 6(5,8) | 0.442 | 0.120* |
| HADS-Anxiety [M(P25,P75)] | 5.33(3,7) | 6(3,7) | 0.886 | 0.023* |
| HADS-Depression [M(P25,P75)] | 6.78(5,8) | 6.5(5,8) | 0.713 | 0.058* |

Note:

1. *Rank-biserial correlation coefficient(rᵣ₆); †Absolute Risk Difference(ARD);
2. Data formats: Values are displayed as N (%) for frequencies and percentages, or M (P25, P75) for medians and interquartile ranges.

Table S2. Baseline Characteristics of Analysed, Excluded, and Lost-to-Follow-up Patients

| **Variable** | **Analysed(n=227)** | **Excluded(n=14)** | **Lost to follow-up(n=11)** |
| --- | --- | --- | --- |
| Age [years, M(P25,P75)] | 66.28(60,72) | 66.5(62.25,72.25) | 68(60,73) |
| Gender [n(%)] |  |  |  |
| Male | 47(20.7) | 3(21.43) | 2(18.18) |
| Female | 180(79.3) | 11(78.57) | 9(81.82) |
| Ethnicity [n(%)] |  |  |  |
| Other | 43(18.94) | 2(14.29) | 2(18.18) |
| Han | 184(81.06) | 12(85.71) | 9(81.82) |
| Residence [n(%)] |  |  |  |
| Rural | 144(63.44) | 9(64.29) | 7(63.64) |
| Urban | 83(36.56) | 5(35.71) | 4(36.36) |
| BMI [kg/m², M(P25,P75)] | 24.95(22.35, 27.68) | 24.6(22.23,26.67) | 25.1(21.8,28.3) |
| Smoking History [n(%)] |  |  |  |
| No | 194(85.46) | 12(85.71) | 9(81.82) |
| Yes | 33(14.54) | 2(14.29) | 2(18.18) |
| Alcohol Use [n(%)] |  |  |  |
| No | 165(72.69) | 10(71.43) | 8(72.73) |
| Yes | 62(27.31) | 4(28.57) | 3(27.27) |
| Sleep Quality [n(%)] |  |  |  |
| Poor | 100(44.05) | 6(42.86) | 5(45.45) |
| Good | 127(55.95) | 8(57.14) | 6(54.55) |
| Comorbidities [n(%)] |  |  |  |
| None | 75(33.04) | 5(35.71) | 4(36.36) |
| Present | 152(66.96) | 9(64.29) | 7(63.64) |
| Prior Knee Surgery [n(%)] |  |  |  |
| No | 182(80.18) | 11(78.57) | 9(81.82) |
| Yes | 45(19.82) | 3(21.43) | 2(18.18) |
| Disease Duration [n(%)] |  |  |  |
| ＜5years | 71(31.28) | 5(35.71) | 4(36.36) |
| 5-10 years | 122(53.74) | 7(50.00) | 4(36.36) |
| ＞10 years | 34(14.98) | 2(14.29) | 3(27.27) |
| Preoperative Pain [M(P25,P75)] | 6.18(6,6) | 6(6,6) | 6(6,6) |
| Anesthesia Type [n(%)] |  |  |  |
| Combined Spinal-Epidural Anesthesia | 111(48.9) | 7(50) | 5(45.45) |
| General Anesthesia | 116(51.1) | 7(50) | 6(54.55) |
| Patient-Controlled Analgesia Pump [n(%)] |  |  |  |
| No | 40(17.62) | 2(14.29) | 3(27.27) |
| Yes | 187(82.38) | 12(85.71) | 8(72.73) |
| Adjunctive Meds [n(%)] |  |  |  |
| No | 149（65.64） | 9（64.29） | 7（63.64） |
| Yes | 78（34.36） | 5（35.71） | 4（36.36） |
| Surgical Side [n(%)] |  |  |  |
| Left | 102(44.93) | 6(42.86) | 4(36.36) |
| Right | 125(55.07) | 8(57.14) | 7(63.64) |
| PCS Score [M(P25,P75)] | 30.27(24,37) | 30.5(23.5,36.25) | 26(23,35) |
| FCIQ Score [M(P25,P75)] | 6.73(5,8) | 6(5,8) | 6(5,8) |
| HADS-Anxiety [M(P25,P75)] | 5.33(3,7) | 6(3,7) | 6(3,8) |
| HADS-Depression [M(P25,P75)] | 6.78(5,8) | 6.5(5,8) | 7(6,8) |

Table S3. Mean (Standard Error) of Intercept and Slope for Each Latent Class

|  | Moderate-High Persistent Pain Group (n=101) | | | | Moderate-Low Rapid Relief Group (n=126) | | | |
| --- | --- | --- | --- | --- | --- | --- | --- | --- |
|  | *M* | *SE* | *t* | *P* | *M* | *SE* | *t* | *P* |
| *I* | 6.956 | 0.073 | 95.288 | ＜0.001 | 5.631 | 0.041 | 137.341 | ＜0.001 |
| *S* | -0.494 | 0.016 | -30.875 | ＜0.001 | -0.631 | 0.018 | -35.056 | ＜0.001 |
